# Direct molecular-level near-field plasmon and temperature assessment in a single plasmonic hotspot

**DOI:** 10.1038/s41377-020-0260-9

**Published:** 2020-03-09

**Authors:** Marie Richard-Lacroix, Volker Deckert

**Affiliations:** 1 Leibniz Institute of Photonic Technology (IPHT), Albert-Einstein-Strasse 9, D-07745 Jena, Germany; 2 Institute of Physical Chemistry and Abbe Center of Photonics, University of Jena, Helmholtzweg 4, D-07743 Jena, Germany

**Keywords:** Nanophotonics and plasmonics, Sub-wavelength optics, Raman spectroscopy

## Abstract

Tip-enhanced Raman spectroscopy (TERS) is currently widely recognized as an essential but still emergent technique for exploring the nanoscale. However, our lack of comprehension of crucial parameters still limits its potential as a user-friendly analytical tool. The tip’s surface plasmon resonance, heating due to near-field temperature rise, and spatial resolution are undoubtedly three challenging experimental parameters to unravel. However, they are also the most fundamentally relevant parameters to explore, because they ultimately influence the state of the investigated molecule and consequently the probed signal. Here we propose a straightforward and purely experimental method to access quantitative information of the plasmon resonance and near-field temperature experienced exclusively by the molecules directly contributing to the TERS signal. The detailed near-field optical response, both at the molecular level and as a function of time, is evaluated using standard TERS experimental equipment by simultaneously probing the Stokes and anti-Stokes spectral intensities. Self-assembled 16-mercaptohexadodecanoic acid monolayers covalently bond to an ultra-flat gold surface were used as a demonstrator. Observation of blinking lines in the spectra also provides crucial information on the lateral resolution and indication of atomic-scale thermally induced morphological changes of the tip during the experiment. This study provides access to unprecedented molecular-level information on physical parameters that crucially affect experiments under TERS conditions. The study thereby improves the usability of TERS in day-to-day operation. The obtained information is of central importance for any experimental plasmonic investigation and for the application of TERS in the field of nanoscale thermometry.

## Tip-enhanced Raman spectroscopy: convenient characterization

Experiments involving tip-enhanced Raman spectroscopy (TERS) could benefit from a convenient method to quantitatively characterize the process and thus aid its optimization. TERS is a highly sensitive technique that combines the chemical specificity of Raman spectroscopy with the spatial resolution of tip-based atomic force microscopy. However, evaluating and quantifying the underlying plasmon mechanisms behind the technique is challenging. Now, Marie Richard-Lacroix and Volker Deckert from Jena, Germany, report that simultaneous collection and analysis of Stokes and anti-Stokes Raman signals provide critical information about the plasmon-based TERS hotspot, including the precise spectral position and width of the plasmon resonance and the near-field temperature. TERS experiments performed with a monolayer of mercaptododecanoic acid on a gold substrate confirm the viability of the approach.

## Introduction

**Fig. 1:**
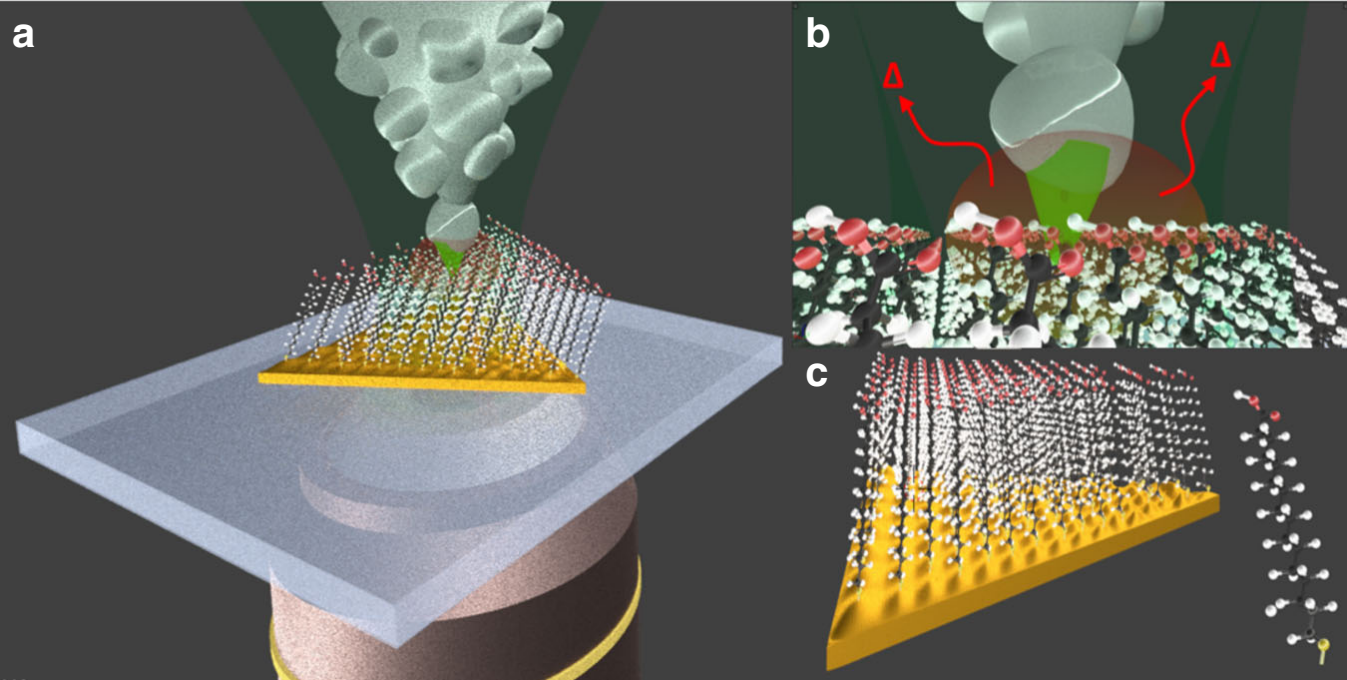
Simplified representation of a TERS setup for a typical exeperiment. **a** Simplified representation of a TERS setup for a typical experiment. Light is focused at the apex of a silver-coated tip situated on top of an ultra-flat gold nanoplate covered by a 16-mercaptohexadodecanoic acid self-assembled monolayer. **b** Zoom on the tip apex where, in the near field, light is confined at the surface of the last particle, where a strong field enhancement occurs due to the formation of a surface plasmon whose energy (with respect to the laser line, which dictates the field enhancement) and spatial full width at half maximum (defining spatial resolution) are unknown. The surface plasmon also generates an a priori unknown temperature rise at the tip apex due to the Joule effect. **c** Zoom in on the 16-mercaptohexadodecanoic acid attached on the gold plate surface through a sulfide bond and a 3D representation of the molecule

Tip-enhanced Raman spectroscopy (TERS) combines the chemical specificity of Raman spectroscopy and the scanning capabilities of atomic probe microscopy (AFM) due to the plasmonic enhancement of a metallic (or metal-coated) tip. As summarized in Fig. 1a, in the far field, light is focused at the tip apex by a microscope objective, which leads to the formation of a surface plasmon confined to the last particle of the tip apex. The surface plasmon, which originates from the collective oscillation of the electrons in the conduction band of the metal, causes an immense enhancement of the electromagnetic field at the tip/sample junction, which enables probing the Raman signal from a nano- and even sub-nanometre-scale area^1–3^. Since its early development in the beginning of the 2000s^4^, TERS has established itself as an indispensable tool for nanoscale surface characterization in various fields^5^. Despite its tremendous potential for detailed structural investigation of nanomaterials, the method’s general utilization is limited by the complexity of the obtained spectral information and the lack of knowledge about crucial parameters affecting day-to-day experiments.

The plasmon resonance position and width are at the very heart of any surface-enhanced Raman spectroscopy (SERS) and TERS measurement. The tuning of the laser, with respect to the plasmon resonance, dictates the enhancement factor and, at the extreme, the presence/absence of any enhanced Raman signal arising from the plasmonically active substrate (here, the tip in Fig. 1a, b). Experimental evaluation of the plasmon resonance that is experienced at the molecular scale in the optical near field is, however, among the main and most critical challenges in the field. For a typical SERS substrate, the solution may be as simple as measuring UV-vis absorption or dark-field spectra, to obtain a global response of the system in the optical far field. Nevertheless, macroscale far-field characterization is intrinsically an average over a large area that does not correspond to the individually and locally experienced electric field^6,7^. The near-field optical response is, however, strongly localization-dependent and its amplitude is directly related to atomic-scale features at the surface of the plasmonic structure^1,8^. Techniques that have been shown to efficiently spatially map the plasmon energy of SERS substrates with sub-nanometre-scale resolution include scattering scanning near-field optical microscopy^9^, (although the possibility of artefacts and misinterpretation remains high)^10^, photoemission electron microscopy^11^, and electron energy loss spectroscopy^12^. This spatial mapping is required to investigate the impact of atomic-scale features on plasmonic and molecular response interpretation^1,8^.

In TERS, however, technical limitations due to the intrinsic conical shape of the tip complicate and even prevent any accurate experimental evaluation of the tip’s plasmonic activity. For tip optimization and basic characterization, especially when considering often substantial tip-to-tip variability^6^, there is an urgent requirement for an accessible, non-destructive, and fast method that enables routine evaluation of tip activity. This kind of evaluation is particularly important, considering the limited lifetime/shelf life of some plasmonic tips, particularly when they are composed of silver^13^. Such a method is also key to enabling any repeatable and reliable quantification, because it defines the relative enhancement of Raman modes.

Additional local heating induced by plasmonic activity is another fundamental but often neglected parameter of any TERS experiment (Fig. 1b). In the past few years, thermo-plasmonics, based on light-to-heat conversion due to the Joule effect caused mainly by light absorption of plasmonic particles^14^, have led to a broad range of applications. In particular, photothermal therapeutics (including cancer therapy)^15^, controlled drug delivery^16^, surface property modulation^17^, photoacoustic imaging^18^, protein digestion^19^, optothermal trapping, and optofluidics^20^ should be mentioned here. From a spectroscopic point of view, near-field heating is one of the most unacknowledged and difficult parameters to evaluate but is nevertheless an integral part of any plasmonic experiment^21,22^. In general, near-field heating at the molecular scale is expected to impact the reactivity and kinetics of transformations^23^, phase transitions and degradation^24^, and diffusion and spatial orientation/reorientation of the molecules under investigation^25^, which clearly impacts data interpretation. Nevertheless, if plasmonic heating can be properly controlled and applied, TERS probes have the potential to act as spatially localized nanometre-sized heat sources for spatially controlled heat-induced system modifications.

Gaining knowledge on the amplitude, spatial profile distribution, and dissipation time of the near-field-generated heat is as complex as the accurate evaluation of the plasmon resonance^14,22^. Heat generation is related to the amplitude of the plasmon, its spatial confinement, and the incident laser power, just to mention a few factors^14^. In addition, Baffou et al.^26^ showed that the spatial heat distribution can also differ significantly from the spatial hotspot distribution. Simulations of an accurate near-field temperature rise therefore require prior knowledge of parameters that, in TERS, are intrinsically unknown. Most studies of this parameter in SERS, to date, have involved a combination of theoretical investigations and far-field responses measured with techniques such as thermal microscopy^27^, fluorescent imaging^28^, conductance measurements^29^, and, more recently, photothermal heterodyne imaging combined with SERS and scanning electron microscopy^30^. Temperature changes as small as a few tenths of a degree^29^ and up to over 400 °C^30^ have been reported.

Here we address the above-mentioned issues summarized in Scheme 1B, namely the precise and nanoscale quantification of plasmon resonance and near-field heating, by a straightforward simultaneous detection of the Stokes and anti-Stokes signals probed in the TERS experiment. The procedure developed here implements a method presented by Brolo et al.^31–33^ in the context of single-molecule SERS. This approach enables extracting and modelling the plasmon resonance and the near-field temperature at the hotspot site, i.e., with the help of solely the molecules contributing to the Raman signal. For this demonstration, we used the TERS spectra of 16-mercaptohexadodecanoic acid, self-assembled as a monolayer, on an ultra-flat, thin gold substrate (Scheme 1C). In the second part of this study, the results are combined to discuss the impact of temperature on nanoscale surface morphology changes of the tip and to probe the existence of atomic-scale protrusions that relate to the spatial resolution through the appearance of blinking lines.

## Results

### Plasmon resonance and near-field temperature

**Fig. 2:**
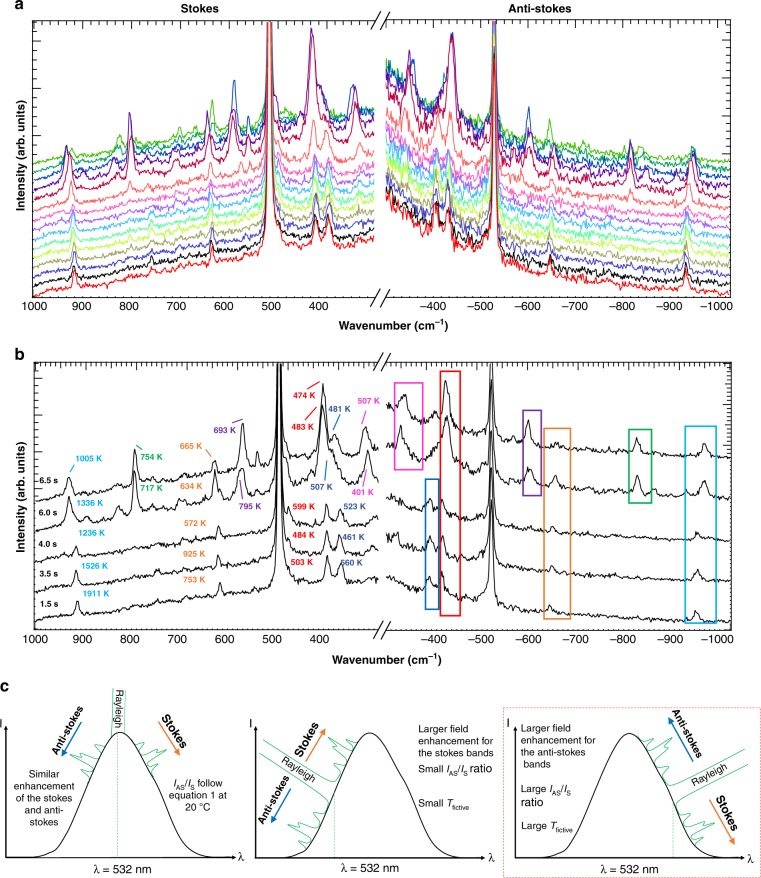
TERS Stokes and Anti-Stokes experiments in relation to near field heating and field enhancement. **a** Series of Stokes and anti-Stokes TERS spectra simultaneously detected (*t*_acq_: 500 ms/spectrum. **b** Selected spectra with a “fictive temperature” calculated by the Stokes and anti-Stokes ratios according to Eq. 1, i.e., by purposely (i.e., wrongly) assuming that only a Boltzmann thermal population distribution contributes to the Stokes and anti-Stokes relative intensities. **c** Schematic representation of the three main scenarios for Stokes and anti-Stokes symmetric or anti-symmetric enhancement based on the approximation that only electromagnetic enhancement is affecting the respective intensities

Figure 2a shows the 300–1000 cm^−1^ Stokes and anti-Stokes spectral region of 16-mercaptohexadodecanoic acid self-assembled on a single ultra-flat gold plate. Unless otherwise mentioned, all TERS experiments presented here were in AFM contact mode, with a laser power at the sample of 0.1 mW and an acquisition time of 500 ms. Furthermore, all spectra presented in Fig. 2a originate from single-point measurements; i.e., the position of the tip on the plate remained unchanged during the experiment. The band centred at ~ 920 cm^−1^ is due to the stretching of the C-COO group when it is deprotonated^34^. The presence of a band at 630 cm^−1^, as well as the absence of a band in the region of ~ 700 cm^−1^, is associated with the gauche conformer of the S-C-C bond^35^, whereas the doublet in the 400 cm^−1^ region is likely due to C-C-O bonding^34^. The absence of a strong signal from 750 to 780 cm^−1^, the C-N^+^ stretching region of ethyltrimethylammonium bromide, strongly suggests the absence of synthesis residues on the gold plate^36^. Figure 2a represents a typical time series of spectra measured at one specific location, where the appearance of new bands in the later stage is quite obvious. This information will be reviewed in the Discussion section (vide infra). The signal-to-noise ratio is impressively large for almost all bands even in the anti-Stokes region, allowing band-fitting procedures to reliably extract band intensities and positions at specific times. For the purpose of visualization, the offset of the anti-Stokes spectra has been adjusted to match that of the Stokes spectra; otherwise, no further scaling or smoothing procedure has been applied to the data. Under ambient conditions and without considering any inhomogeneous enhancement due to, e.g., resonance or charge transfer effects, one can approximate that the scattering cross-sections for the ground and exited vibrational states are equal. The thermal population could then be described by the Boltzmann distribution, which predicts an exponential decrease in the anti-Stokes/Stokes intensity ratio as a function of the wavenumber according to Eq. 1^37^.

1 $$\rho = \frac{{I_{\mathrm {AS}}}}{{I_{\mathrm {S}}}} = \left( {\frac{{\bar v_{\mathrm {laser}} - \bar \nu _R}}{{\bar \nu _{\mathrm {laser}} + \bar \nu _R}}} \right)^3{\it{\mathrm {exp}}}\left( {\frac{{ - hc\bar \nu _R}}{{kT}}} \right)$$

where *I*_A_ and *I*_AS_ are the respective Stokes and anti-Stokes intensities, and $\bar \nu _{\mathrm {laser}}$ and $\bar \nu _R$ represent the energy of the incident laser line and of the Raman mode, respectively, given in cm^−1^; *h* is the Planck constant, *c* is the speed of light in vacuum, *k is* the Boltzmann constant, and *T is* the temperature. The presence of a strong anti-Stokes signal with a comparable signal-to-noise ratio would indicate a high population of the excited vibrational state, i.e., a high temperature. At room temperature, for instance, Eq. (1) predicts that the anti-Stokes vs. Stokes ratio of the 920 cm^−1^ band should be ~ 0.008, which is much lower than any ratio for the spectra shown in Fig. 2b.

Figure 2b shows the “fictive temperatures” (*T*_fic_) quantified individually and after calibration for the optical response of the setup, using Eq. 1 for all bands of each spectrum. Noticeably, for a given spectrum, all vibrational modes refer to different *T*_fic_, which is clearly unphysical, and thus shows that the band ratios are not only due to temperature effects. In all cases, the *T*_fic_ is high and even reaches impressive values of almost 2000 K (see also Supplementary Fig. S1 of the Supporting Information). This temperature is well above any damage threshold of organic materials. Any damage would also be easily exposed in the time series. As such, damage was not observed, which also indicates a non-thermal contribution to the band ratios. The term “fictive temperatures” is used to specifically emphasize that under SERS/TERS conditions, a temperature cannot be quantified via the Boltzmann equation alone and the Stokes/anti-Stokes ratio depends on more factors other than exclusively the temperature. For a single vibrational mode, *T*_fic_ also varies as a function of time by as much as 1000 K. Most importantly, *T*_fic_ is clearly increasing as a function of wavenumbers, a trend that also remains over time (although the signal amplitude itself changes in both spectral ranges).

To the best of our knowledge, the high intensity of the anti-Stokes spectral range was originally reported by Kneipp et al. and discussed as an optical pumping effect, i.e., by creating a considerable population at the first exited vibrational state due to high cross-section, introduced by the SERS process, through a “chemical effect”^38^. The concept was later challenged and eventually attributed to asymmetric field enhancement of the scattered field^31,39^. In non-resonant Raman conditions, when neglecting any charge transfer effects, the field enhancement is mainly attributed to the electromagnetic mechanism. Three extreme scenarios that rely on the relative positioning of the incident line with respect to the plasmon resonance can be envisioned, as summarized in Fig. 2c. The *E*^4^ approximation commonly used suggests that the field enhancement is identical, or at least similar, for both the incident laser line and scattered field Raman signal. Under optimal conditions, the plasmon is centred at the excitation wavelength in the context of this study at 532 nm. In the experimental reality, as shown schematically in the left panel of Fig. 2c, when large spectral ranges are considered, the *E*^4^ approximation only holds for a broad plasmon resonance excited at the peak position, leading to an equivalent enhancement of the Stokes and anti-Stokes spectral ranges. In such a case, the experimental TERS anti-Stokes/Stokes ratio would be roughly equivalent to an anti-Stokes/Stokes ratio measured at room temperature in the far field (i.e., considering the thermal distribution of Eq. 1 and neglecting a temperature rise in the near field). The centre and right panels of Fig. 2c represent simplified situations where this symmetry is absent and, in particular, the incident laser line (532 nm) is not situated directly at the centre of the plasmon resonance. The Stokes part of the spectrum is more (centre panel) or less (right panel) enhanced with respect to the anti-Stokes part so that the fictive temperature is either higher (centre) or lower (right) than that in the symmetric case. When comparing the three model scenarios with the quantified *T*_fic_ in Fig. 2b, one can conclude that the surface plasmon experienced by the molecules at the apex of the tip used for this experiment is best represented by the right panel.

Consequently, the relative band intensities and thus the anti-Stokes/Stokes ratios (and thus all *T*_fic_ values quantified in Fig. 2b) on top of the Boltzmann distribution of the vibrational levels are convoluted with the wavelength-dependent plasmonic enhancement, which can be described by a Lorentzian function. Starting from Lombardi and Birke^40^, who described the SERS intensity as the product of a localized surface plasmon, CT, and Raman resonance, the Brolo group^31–33^ developed a model linking the Stokes/anti-Stokes ratio quantified for any Raman mode to the localized plasmon resonance. These equations are further adapted here to extract the near-field temperature rise, i.e., the temperature de facto experienced by the molecules in the near field. Here we use the availability of multiple bands with a sufficiently high signal-to-noise ratio to expand Brolo’s formalism into a series and further add temperature as a free parameter. As we have purposely chosen a non-resonant system to avoid further complexity, here we only consider electromagnetic field-enhancement mechanisms. A detailed description of our model in comparison with the Brolo studies is provided in the Supporting Information.

The model and the respective equations applied in this study, i.e., Eqs. 2 to 4, link *K*, the anti-Stokes/Stokes intensity ratios measured in TERS (*I*_AS,TERS_ and *I*_S,TERS_) normalized by the ratios obtained from a reference experiment (*I*_AS,ref_ and *I*_S,ref_)—measured from cysteine crystals in the far field—with a Lorentzian description of the electromagnetic enhancement (*K*_EM_) as described by Brolo et al.^31–33^. Here, the thermal distribution in the near field is added in the final fit as Eq. 4:

2 $$K = \frac{{I_{\mathrm {AS,TERS}}}}{{I_{\mathrm {S,TERS}}}}\big/\frac{{I_{\mathrm {AS,ref}}}}{{I_{\mathrm {S,ref}}}}$$

3 $$K_{\mathrm {EM}} = \left[ {\frac{{\left( {\overline \nu _{\mathrm {plasmon}} - \overline \nu _{\mathrm {laser}} + \overline \nu _R} \right)^2 + \left( {\frac{1}{2}{\mathrm{\Gamma }}} \right)^2}}{{\left( {\overline \nu _{\mathrm {plasmon}} - \overline \nu _{\mathrm {laser}} - \overline \nu _R} \right)^2 + \left( {\frac{1}{2}{\mathrm{\Gamma }}} \right)^2}}} \right]$$

4 $$\mathop {\sum}\limits_i {K_i = \frac{{\rho _{\mathrm {exp},i}}}{{\left( {\frac{{\overline \nu _{\mathrm {laser}}\; -\; \overline \nu _{R,i}}}{{\overline \nu _{\mathrm {laser}}\; + \;\overline \nu _{R,i}}}} \right)^3 {\mathrm {exp}}\left( {\frac{{ - ch\overline \nu _{R,i}}}{{kT}}} \right)}}} = \mathop {\sum}\limits_i {\left( {\frac{{\left( {\overline \nu _{\mathrm {plasmon}}\; - \;\overline \nu _{\mathrm {laser}} + \overline \nu _{R,i}} \right)^2 + \left( {\frac{1}{2}{\mathrm{\Gamma }}} \right)^2}}{{\left( {\overline \nu _{\mathrm {plasmon}}\; - \;\overline \nu _{\mathrm {laser}} - \overline \nu _{R,i}} \right)^2 + \left( {\frac{1}{2}{\mathrm{\Gamma }}} \right)^2}}} \right)^2}$$

where $\bar \nu _{\mathrm {laser}}$ is the laser excitation energy, $\bar \nu _{\mathrm {plasmon}}$ is the resonance frequency of the plasmon, $\bar \nu _{R,i}$ is the vibrational energy of a specific Raman mode *i* (extracted from Fig. 1a), and *Γ* is a damping parameter for the plasmon resonance. The latter is proportional to the full width at half maximum (FWHM). Again, *c* is the speed of light in vacuum, *h* is the Planck constant, *k* is the Boltzmann constant, *T* is the near-field temperature that appears in the equation as the temperature difference with the bulk reference measured in the far field at 20 °C, and *ρ*_exp,*i*_ is the anti-Stokes/Stokes ratio experimentally evaluated from the TERS measurement for mode *i*. Equations (3) and (4) are based on the approximation that only electromagnetic contributions to the field enhancement are preserved by normalization with a reference.

**Fig. 3:**
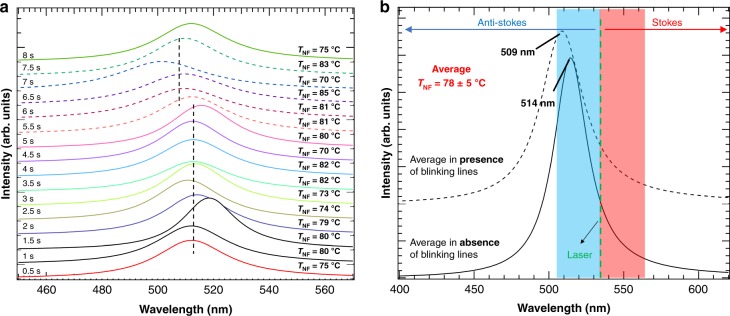
Quantitative temporal study of Plasmon resonance and near-field heating. **a** Calculated plasmon frequency and temperature for every Raman spectrum measured over time (indicated in the figure) and corresponding to the spectra presented in Fig. 1A. **b** Calculated average of the plasmon resonance and average temperature for the TERS spectra in the absence (full curve) and presence (dashed curve) of blinking lines. The blue and red highlights show the limits of −1000 cm^−1^ and +1000 cm^−1^ (anti-Stokes and Stokes Raman signals, respectively) measured experimentally

Using Eqs. (2) to (4), assuming that the plasmonic activity can be approximated by a single Lorentzian line in the absence of resonance conditions, the plasmon resonance corresponding to each spectrum in Fig. 2a can be quantified independently (Fig. 3a). This plasmon band only represents the plasmon resonance experienced by the molecules located directly under the tip that actually contribute to the Raman signal in a time interval of 500 ms. Here, all resonances are centred at a frequency lower than that of the incident laser wavelength (532 nm) with a FWHM that is quite narrow (~ 2300 cm^−1^, corresponding to a dephasing time of ~30 fs; see Supplementary Fig. S2 in the Supporting Information)^41^. Nevertheless, it is still much broader than the FWHM quantified by dos Santos et al.^33^ in the context of single-molecule SERS using a similar strategy (~500 cm^−1^). Under these conditions, any small shift of the plasmon frequency would have drastic effects on the Stokes and anti-Stokes intensities. Obviously, a satisfying signal-to-noise ratio from the anti-Stokes spectral range for at least three bands (for the three unknowns in Eq. 3) is required to properly extract the plasmon band. The absence of any anti-Stokes signal for several bands when measuring with different tips or even in the course of the experiment with the same tip (see Supplementary Fig. S3 of the Supporting Information, where the anti-Stokes spectral range is not following that of Fig. 3c) is a strong indication of a red- or blue-shift of the plasmon frequency in comparison with that in Fig. 2 (vide infra). In line with this consideration, our recent study showed that the plasmonic response of our evaporated silver island tips is largely dominated by the single dipole of the particle composing the tip apex, which furthermore strongly depends on its specific morphology^6^. With a plasmon resonance of such a narrow width, it is thus expected that different tips must have different resonance frequencies that will be reflected in the Stokes vs. anti-Stokes intensity ratios. This expectation also justifies that, at least in our experience, some tips are considered more “active” than others.

In addition to the plasmon parameters, the near-field temperatures are intrinsically available from Eq. (4) and, consequently, in Fig. 3a, the corresponding temperatures for each single spectrum (and in Fig. 3b, the average temperature for the entire experiment) are listed. All temperature values lie within the same range with an average of 78 ± 5 °C. This increase in temperature is also in line with the previously mentioned disappearance of TERS signals after a few seconds spent at a single location on the sample. Desorption and diffusion of the self-assembled monolayer on the gold surface becomes important at this temperature^42,43^.

Several groups have attempted to quantify the near-field temperature rise under TERS conditions. Yue et al.^44^, for instance, used a Si substrate signal mapped with a gold-coated tip and utilized the Si band positions to evaluate the near-field temperature. The authors concluded an environment as hot as 250 °C for their experimental conditions. This would be ~100 °C above the damage threshold temperature of the 16-mercaptohexadodecanoic acid self-assembled monolayers investigated here^45^. Large temperature changes of ~100 °C are required for any measurable shift of the Si band to be detected, which is far above the values quantified here and not precise enough to enable an accurate evaluation of the local heating of the molecules under the tip. Here, as no shift in the 520 cm^−1^ Si Raman mode could be quantified within the experimental error (Supplementary Fig. S1), our quantified near-field temperature rise of ~80 °C remains consistent with their conclusions, albeit indicating lower temperatures for our conditions. High temperature was also quantified by Balois et al.^46^, who used Stokes and anti-Stokes relative intensities of carbon nanotube bands measured by terahertz spectroscopy. Their calculation, however, neglected any asymmetry of the enhancement factors, which in the present study is shown to be quite high, even close to the Rayleigh line. Here, for instance, a value of ~65 °C has been quantified for the Si band (originating from the tip) with the help of the anti-Stokes/Stokes ratios (see Supplementary Fig. S1 of the Supporting Information) when considering solely a Boltzmann distribution (Eq. (1)). This is the only signal of the spectra that originates from both the near field and the far field, as plasmonic activity is not a requirement for observing the silicon signal. This Si-extracted value alone thus does not exclusively refer to the near-field environment. Hence, the results need to be carefully confirmed even if the considered Stokes/anti-Stokes are close to the Rayleigh line. It is also worth mentioning that as long as resonant Raman conditions are avoided and no electron transfer occurs from tip to molecule (or vice versa), the chemical nature of the molecules or the specific vibrational modes probed do not play a role.

## Discussion

### Surface morphological characteristics of the TERS tips

The surface morphology, particularly the atomic-scale surface morphology of the silver particles (in the form of picocavities, atomic-scale protrusions, or, from a more general point of view, atomic-scale surface defects) theoretically defines the spatial confinement of the plasmon and thus the spatial resolution of any TERS measurement^1–3,8^. Although spatial resolution in TERS is still an active topic of discussion, several experimental demonstrations of submolecular resolution were made in recent years by different research groups, especially as a result of experimental mapping of immobilized species involving ultra-high vacuum, low-temperature setups^2,3^. Benz et al.^47^ proposed an alternative method to experimentally investigate the surface features responsible for high field confinement. Using a particle on a mirror configuration, they demonstrated that picocavities form (and are destroyed) at the surface of gold particles at cryogenic temperature under laser irradiation. Their main hypothesis is that the so-called persistent lines, in other words, bands that appear in all spectra, appear because of the single dipole resonance of the particle, whereas blinking lines, i.e., strong bands that appeared after laser irradiation originate from picocavity formation that further confines the field, induces a drastic change in selection rules and leads to the appearance of infrared active modes in the Raman spectra^47^. The original concept was recently extended to silver^48^ and gold particles^49^, where it has been shown that plasmonic picocavities are stable for a few seconds under ambient conditions. As our experiment is conceptually similar to the method of Benz et al.^47^, we will discuss the results in view of our access to the plasmon parameters and the temperature.

**Fig. 4:**
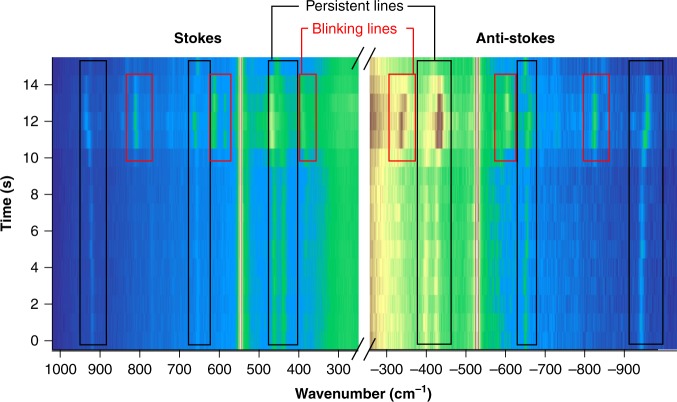
Waterfall plot of the Raman signal for the Stokes and anti-Stokes spectral ranges over time, with a time resolution of 500 ms. The persistent and blinking lines are present in each spectrum in this time frame (black rectangles) and appear for a few seconds (red rectangles), respectively

Figure 4 presents the waterfall plot of the spectra shown in Fig. 2. Clearly, two sets of lines (persistent and blinking lines) can be distinguished and interpreted by analogy with the original study from Benz et al.^47^. The main difference between the two experiments is that in the particle on a mirror configuration of ref. ^47^, the plasmonic particle lies flat on the surface, whereas in our case the plasmonic tip approaches it from the top (Fig. 1). The TERS tips used in our experiment are prepared by physical vapour deposition (PVD) of silver on silicon AFM tips to cover the tip with a silver island film^50^. The tip (or more specifically the last silver particle) is brought into contact with a flat gold surface covered by a self-assembled monolayer of 16-mercaptohexadodecanoic acid. Consequently, the major difference between a particle on a mirror configuration and our experiment is that a free particle would most likely expose a facet to a flat surface to optimize its contact area and minimize the surface energy, whereas the tip will approach the surface via an edge site. The latter assumption is supported by high-resolution transmission electron microscopy (TEM) images of our tip apex that exposed distinct crystalline structures (faces, edges, and corner sites) on the silver particles, which, in the course of the experiment, are brought in direct contact with the surface^51^. Given the complexity and variability of the metallic surface of the TERS probe, a recent study from our group has shown that the system could be simplified by modelling a perfect sphere with atomic-scale protrusions emerging from its surface. This model limits the field to a small area, leading to a sub-nanometre resolution (see Supplementary Fig. S4)^51^ that is in agreement with experimental and theoretical evaluations^1,3,8^.

**Fig. 5:**
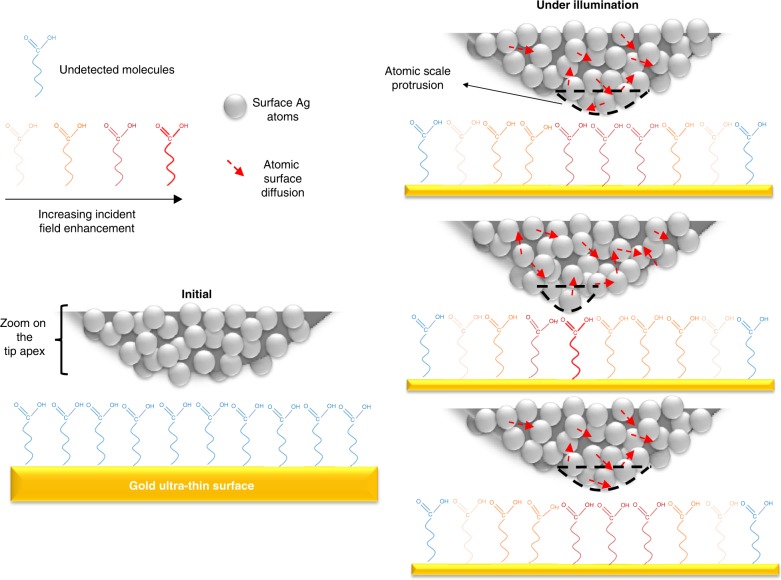
Schematic representation of the last particle at the tip apex and the assumed formation of picocavities (or nanoscale protrusions). **a** Schematic representation of the 16-mercaptohexadodecanoic acid molecules where the colour gradient from light orange to dark red qualitatively represents the field enhancement that they experience and where blue molecules are undetected. The grey spheres are associated with silver atoms at the surface of the tip, and the red arrows schematically represent thermally induced movements. **b** Silver-coated TERS tip approaching the sample prior to illumination. **c** When the tip apex is illuminated and in contact with the surface, a stronger Raman signal arises from molecules directly in contact with the nanoscale protrusions (represented by the dashed black region) (top right). During illumination, a near-field induced temperature rise can induce the surface diffusion of silver surface atoms (red arrows) and a change in the tip apex morphology. After some time, a new atomic-scale protrusion is created, leading to the appearance of blinking lines in Fig. 1 and to an increase in the amplitude of the field experienced by the molecule directly located under the protrusion (middle right). In our experiments, this atomic-scale protrusion is modified within a few seconds again due to the surface diffusion of silver and, in this case, to the reduction of the field enhancement

To relate the peculiarities of the specific atomic structure of the tip and its effect on the spectra, Fig. 5 qualitatively shows how Å- or nanoscale-scale surface protrusions already exist at the surface and how morphological changes during the experiment time can be understood in terms of the proposed dynamic creation and annihilation of picocavities (demonstrated by ref. ^47–49^). By looking at the TEM image of the tip apex, i.e., the last particle of the tip, we can see that the surface contains defects that are partially disorganized in comparison with a crystal facet. The direct contact area of the last particle with the surface is rather small and necessarily occurs through one type of defect or another. Under illumination, the Raman signal theoretically corresponds to an overlap of the single dipole resonance of the particle and of the additional enhancement deriving from further confinement via the presence of picocavities or protrusions^47,51^. The number of molecules contributing to the signal and their relative contribution to this signal define the spatial resolution. As in the present case the same type of molecule covers the entire surface, it is impossible to quantitatively evaluate the lateral resolution solely through experimental results. However, the sudden appearance of blinking lines and the remaining persistent lines (with a stronger signal) point in the direction of sub-nanometre-scale resolution. As some protrusion was initially present at the tip apex, it is unlikely that it was suddenly created, as was the case in previous studies of picocavity formation^47–49^. Nevertheless, laser exposure in resonance with plasmonic modes promotes the diffusion of metal surface atoms, leading to changes in morphology at the tip surface. The local temperature value of ~80 °C quantified above is only ~45 °C lower than the annealing temperature applied for silver island production following PVD deposition^52^, suggesting that the temperature value is high enough to induce minor changes in the particle morphology. Such surface diffusion can be deduced by the comparison of the study by Benz et al.^47^ (whose picocavity has been shown to be stable under cryogenic conditions) and the most recent study from Shin et al.^48^, who showed that their picocavities could only be stable for a few seconds under ambient conditions. In line with the above argument, atomic surface diffusion was demonstrated by Zhang et al.^53^, who showed that morphology changes of their nanometre-scale rough gold surfaces occurred solely under TERS experimental conditions. Stöckle et al.^50^ also showed a significant change in morphology and a blue-shift of the plasmon resonance after a few seconds of annealing silver particles at temperatures as low as 200 °C. Those studies again point in the direction of a plasmonic-activated thermally induced morphological change.

At first glance, the results of this study appear slightly different from those of Benz et al.^47^, who reported an absence of persistent lines in the anti-Stokes spectral region. In their experimental conditions, the relative enhancement due to the picocavity vs. the single dipole resonance of the particle (in the absence of a picocavity) is large (~13 times larger). For protrusion at the surface of particles on the TERS tip, our theoretical study predicts a value of 4 at best^51^. However, the field enhancement caused by the single dipole resonance itself is much higher. Persistent lines could thus be observed regardless of the protrusion size, shape, or orientation. Interestingly, the *T*_fic_ values quantified for some modes in Fig. 2b (and Supplementary Fig. S1 in the Supporting Information) are similar and even higher than those quantified by Benz et al.^47^. This observation highlights the similarities of both studies and suggests that the plasmon band shape (if defined as the slope relating the Stokes and anti-Stokes spectral ranges) is likely to be similar for both studies.

This interpretation is also in line with the results presented in Fig. 3c, where when looking closely at the time evolution of the plasmon band, the series splits into two distributions, i.e., one centred at ~514 nm and one blue-shifted and slightly broader at 509 nm for the last ~30% of the spectra, as indicated in the average spectra of Fig. 3b. For both respective series, the plasmon position remains approximately identical when considering the series of spectra separately (as was the case for the temperature). According to theoretical calculations, the formation of an Å-scale protrusion at the surface of a sphere leads to a slight blue-shift of ~1 to 2 nm^51^. The slight blue-shift of the plasmon resonance shown in Fig. 3b that corresponds to the presence of blinking lines is thus consistent with the presence of a nanoscale protrusion at the surface of our tips during this experiment.

A likely scenario of the temporal evolution of the system is thus presented in Fig. 5. Several factors are considered here, namely (1) the appearance of blinking lines as demonstrated in the literature as being related to protrusions; (2) the annealing effects on the tip, which can change the size and shape of the particles due to the rise in near-field temperature quantified in Fig. 3, which is sufficient for silver surface diffusion, as shown in the literature; and (3) the blue-shift theoretically expected when such protrusions are formed, which is consistent with our calculations.

Due to the elevated temperature at the tip apex, the original protrusion (top of Fig. 5) reorganizes and simultaneously modifies its efficiency to confine the field. This change concurrently corresponds to the appearance of blinking lines, which could be either due to newly activated IR modes^47–49^ or to a suddenly dominant signal arising from one or a few molecules whose orientation in space, or interaction with its neighbour or with the tip itself, differ from the previous “nano-ensemble.” This phenomenon simultaneously increases the signal arising from slightly fewer molecules in comparison to the “persistent lines” situation due to the higher field confinement in space and its higher intensity. Within a few seconds, this protrusion is transformed back to either its original state or a different state with similar properties (bottom left of Fig. 5), where the additional confinement is lost simultaneously with the disappearance of blinking lines. This conclusion is also reflected in our theoretical study related to surface protrusions, where, under resonant conditions, the FWHM of the spatial distribution of the field enhancement slightly changes with the orientation of the atomic feature with respect to the particle surface^51^. In Fig. 5, the concept is explained by the involvement of several atoms that create a small atomic cluster that represents larger or smaller protrusions. It is unclear why such small changes should be accompanied by a sudden modification of the selection rules or preferential expression of a few molecules. However, Supplementary Section S3 (Supplementary Fig. S5) of the Supporting Information uses the symmetrical response of the persistent lines of the Stokes and anti-Stokes spectral ranges to rule out other possibilities such as the appearance of contamination.

**Fig. 6:**
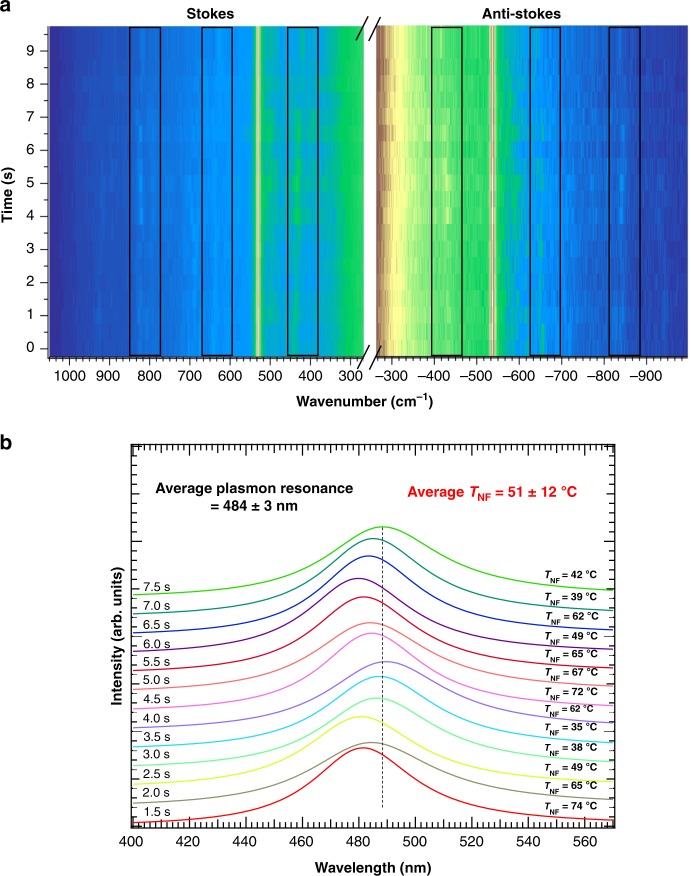
Evolution of the plasmon resonance and near-field heating as a function of time at a 2nd location. **a** Waterfall plot of a series of spectra of a second location on the sample, 3 s after the disappearance of the signal in the first series (Fig. 2a). **b** Plasmon band and near-field temperature quantified from **a** as a function of time. The blue-shift of the plasmon is associated with partial oxidation of the tip, probably accelerated by near-field heating and/or larger scale morphology changes of the tip apex than that represented in Fig. 5

Once the initial protrusion morphology is modified thermally, another protrusion associated with a different field enhancement takes over and so on, until no defect exists at the surface or until oxidation attenuates the tip plasmonic activity. The results observed with the same tip at different times and locations support this conclusion, as shown in Fig. 6a and Supplementary Fig. S3 of the Supporting Information. As seen in the waterfall plot of a second series of spectra measured at a different location three seconds after the measurements shown in Fig. 2 (see also the comparison with Supplementary Fig. S3), some series show blinking lines and others show only a small signal from persistent lines. Notably, the signal-to-noise ratio of the bands decreases over time (see comparison of Fig. 6a with Fig. 4). Applying Eq. (4) to the data sets presented in Fig. 6a clearly shows that the plasmon frequency (Fig. 6b) is significantly blue-shifted for this second series (by ~20 nm), which is consistent with the decaying signal caused by silver oxidation and/or with the systematic blue-shift observed for the SERS silver substrate annealed as a function of time^50^. A similar signal decay has been observed for all tips and can be correlated to both thermally induced morphological changes of the tip and to the use of contact mode, which probably leads to a decrease in the tip’s lifetime. The simultaneously obtained near-field temperature is also significantly cooler than in the original experiment shown in Fig. 3a (by ~25 °C), which is consistent with a much lower absorption coefficient of the plasmon at 532 nm. The much lower Stokes and anti-Stokes spectral intensities obviously lead to a larger fluctuation of the computed near-field parameters. Nevertheless, the results presented here illustrate that the field enhancement variation in time and space modulates the relative spectral intensity contributions of the spectra.

In summary, we succeeded at quantitatively extracting the plasmon resonance parameters and the near-field temperature in a TERS hotspot using simultaneously detected Stokes and anti-Stokes Raman signals. The position of the detected plasmon resonance, here at lower wavelengths than that of the laser line used for Raman excitation, explains the excellent signal-to-noise ratio observed in the anti-Stokes range. The obtained near-field temperatures of ~80 and 55 °C for two consecutive series at an incident laser power of 0.1 mW is consistent with a surface diffusion-based restructuring of the tip morphology, leading to noticeable changes in the spectra, i.e., an increase in the signal amplitude, the frequent appearance of blinking lines and the gradual decrease in the signal amplitude. The decrease and eventual loss of detectable signals following a few seconds of laser illumination and the reappearance of the signal at other locations also strongly suggest that molecules are diffusing out of the probed area, most likely due to the local temperature rise. The appearance of blinking lines accompanied by a blue-shift of the plasmon resonance strongly indicates the presence of nanoscale protrusions that dominate the TERS enhancement, consequently linked to a spatial resolution corresponding to a few molecules at most.

The method is an asset to any day-to-day TERS experiments, as the actual conditions to which the molecules are submitted from one experiment to the next can now be investigated directly, in real time, and at the sample scale. Hence, we believe that this study will facilitate exploring the consequences of near-field optical characteristics on molecular activity and characterizing (and optimizing) plasmonic activity. Parameters obtained via the proposed method will likely help to improve the accuracy of theoretical models, which are intrinsically linked to the spatial field intensity distribution and precise positioning of the plasmon resonance for near-field temperature evaluation. Thus, the procedure can be expected to provide new insights into the experimental parameters playing crucial roles concerning both static and dynamic aspects influencing the plasmon resonance and near-field heating. To the best of our knowledge, no other accessible methodology opens up such a wealth of information on plasmonic activity during a typical TERS experiment.

## Materials and methods

Gold nanoplates were prepared according to a procedure described in detail previously^54^ and transferred onto glass substrates (previously cleaned by immersion in a 3 : 1 HNO_3_/H_2_O_2_ 30% mixture for 2 h, followed by cleaning with deionized water). Self-assembled monolayers of 16-mercaptohexadodecanoic acid were produced by immersing the gold plate substrates for 8 h in a 1 mM 16-mercaptohexadodecanoic acid (Sigma-Aldrich) ethanol solution (HPLC grade, Carl Roth) followed by 10 min immersion in ethanol. The substrate was then dried and kept under an argon atmosphere until usage. An AFM topography image of the plate is provided in Supplementary Fig. S6.

The TERS setup and the detailed procedure for TERS experiments were described in detail in a previous publication^55^. In summary, the system used a 532 nm laser excitation (Cobolt 04-01 series, Sweden) with a power of 100 µW at the sample and consisted of a Raman spectrometer (SP300, Princeton Instruments, USA) equipped with a CCD camera (PIXIS400, Princeton Instruments coupled to an AFM head (NanoWizard 2, JPK, Germany) mounted on an inverted microscope with a ×100 oil-immersion objective (NA 1.30, Olympus, Japan). Ag-coated AFM tips are first positioned in the focus of the laser spot by optimizing the amplitude of the silicon signal using the tip scanner. The sample scanning option is used to control the sample position during the experiments to preserve the tip/laser focus positioning. A full set of 532 nm volume Bragg notch filters (OptiGrate, USA) was used to filter the Rayleigh line, while giving access to low-wavenumber Stokes and anti-Stokes Raman bands. Other experimental conditions are mentioned directly in the text. The Stokes and anti-Stokes relative intensities were calibrated by Raman spectra of polycrystalline cysteine measured at room temperature. The actual Stokes/anti-Stokes ratios were matched to the relative intensity ratios predicted by Eq. (1). The deviation, quantified as a function of wavenumbers (in cm^−1^), was used as an instrument correction parameter to adjust the Stokes/anti-Stokes ratio in the TERS experiments. Intensities and band positions were quantified by fitting each band with a Lorentzian function with the help of IGOR Pro 7 software (WaveMetrics, USA). The plasmon frequency and width, as well as the near-field temperature, were evaluated with the help of Eq. (4) programmed in Mathematica (Wolfram, USA) software. Minimal fit constraints were applied (0 < FWHM < 5000 cm^−1^, 17,000 < $\bar \nu _{res}$ < 21,000 and 0 < *T* < 400 K) so that the model was free to explore a large range of solutions.

## Supplementary information

Supplementary Material.
