# Supplementary material for: Direct molecular-level near-field plasmon and temperature assessment in a single plasmonic hotspot
Source: Light Sci Appl. 2020 Mar 9;9:35. doi: 10.1038/s41377-020-0260-9 (PMC7061098; doi:10.1038/s41377-020-0260-9)
Supplement: Supplementary file 1 — Supplementary Material. [file 41377_2020_260_MOESM1_ESM.docx]

**Direct molecular level near-field plasmon and temperature assessment in a single plasmonic hot spot**

**Supporting Information**

*Marie Richard-Lacroix^1,2^ and Volker Deckert*^1,2^*

1. Leibniz Institute of Photonic Technology (IPHT), Albert-Einstein-Straße 9, D-07745 Jena, Germany
2. Institute of Physical Chemistry and Abbe Center of Photonics, University of Jena, Helmholtzweg 4, D-07743 Jena, Germany

*corresponding author: [volker.deckert@leibniz-ipht.de](mailto:volker.deckert@leibniz-ipht.de)


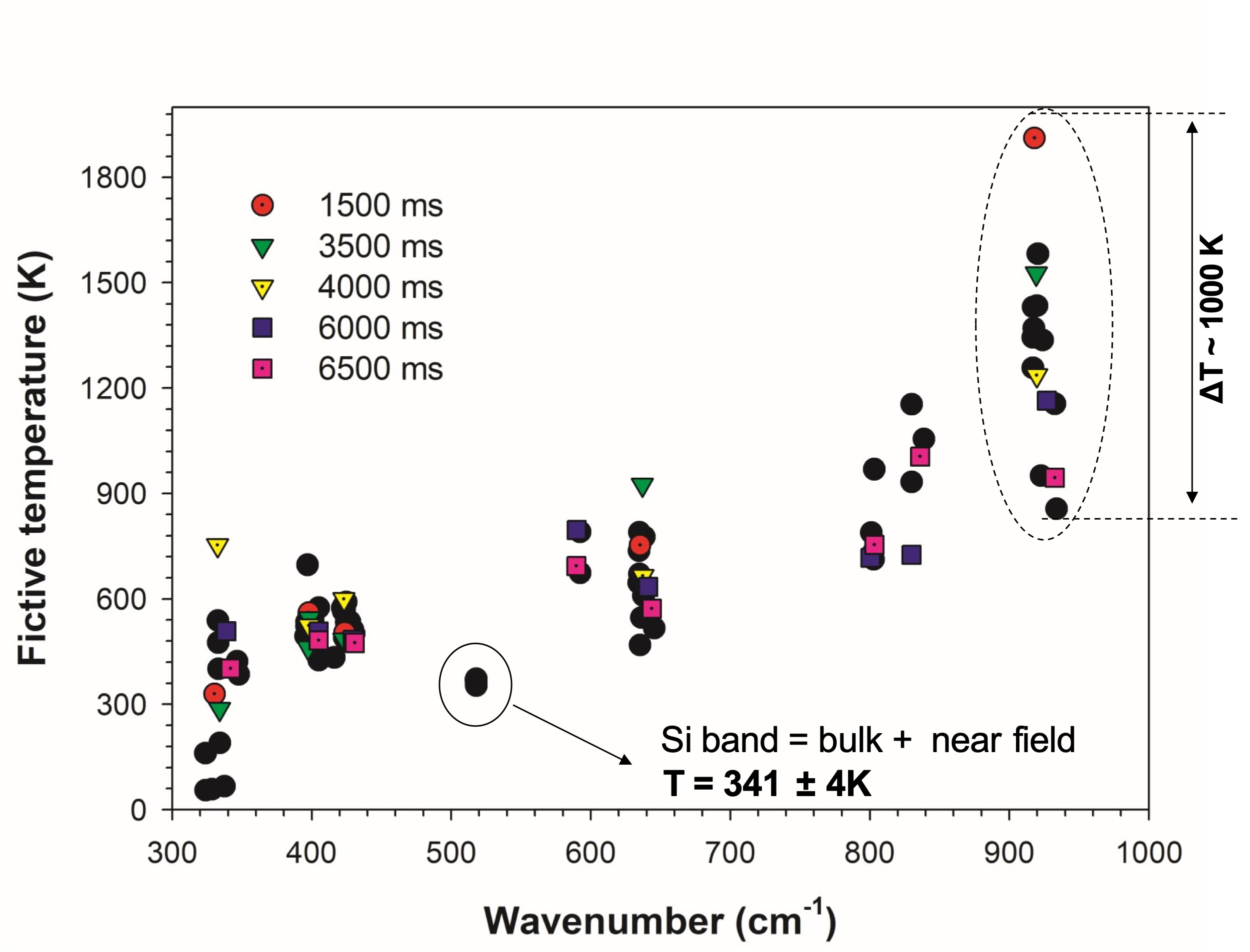


**Figure S1.** Fictive temperature quantified for all bands of the spectra shown in Figure 1A. The colored points are specifically associated to the series of spectra shown in Figure 1B. The black points represent all other spectra of the series.


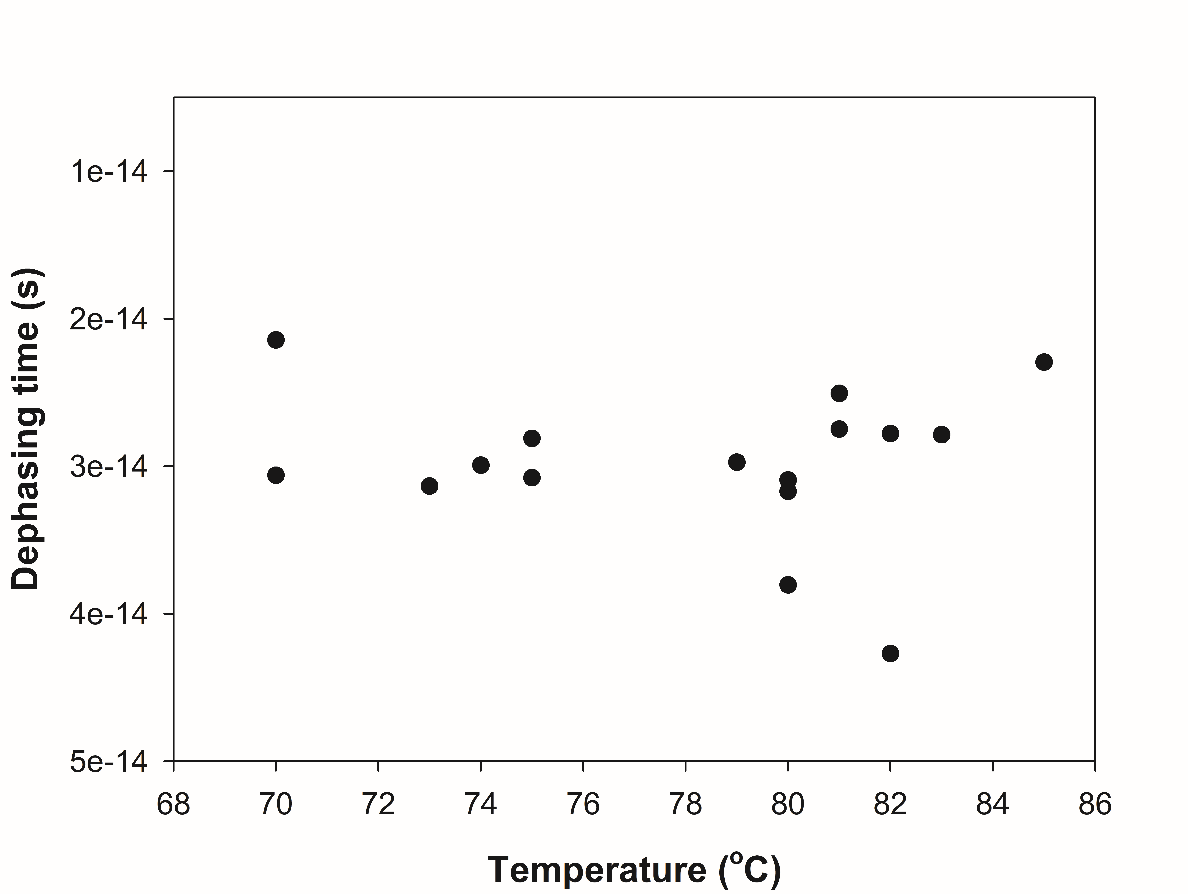


**Figure S2.** Dephasing time calculated of the plasmon resonance quantified from the full width at half maximum of the plasmon band quantified in Figure 3A of the main manuscript from 2ħ/FWHM, as suggested in Ref. 41 of the main text and plotted as a function of the quantified near-field temperature. The plasmon dephasing time is situated in the femtosecond range and appears constant, in the near-field temperature range investigated.

**
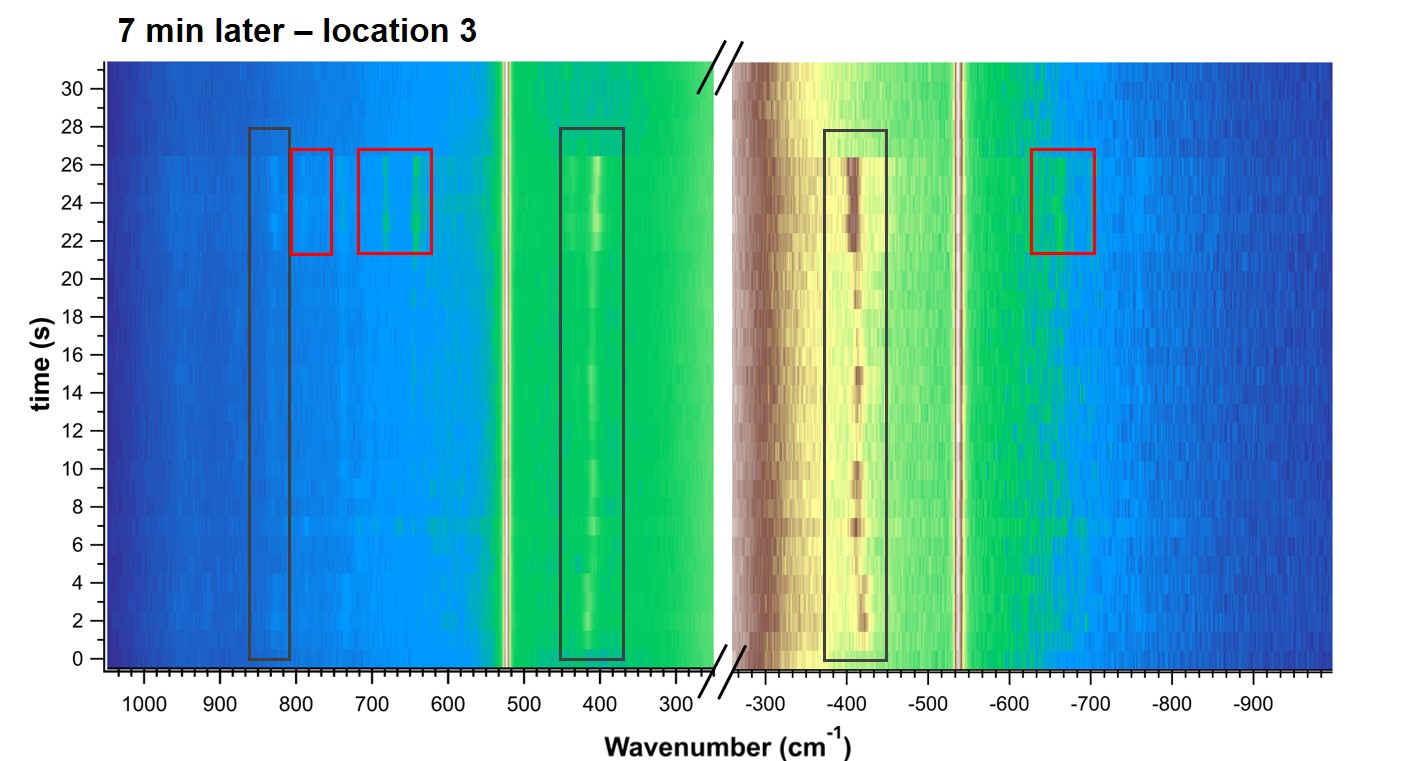
**

**Figure S3.** Waterfall plot of the TERS spectra measured as a function of time (time per spectra of 500 ms), measured at a different location on sample 7 minutes after the series shown in Figure 2C.

**Section S1. Tip apex morphology**

**Figure S4.** Schematic detailed representation of a tip produced via evaporative silver coating. When zooming at the apex of the last particle at the apex of the tip, TEM results shows that the particle is mainly composed of polycrystalline silver and a surface layer composed of defects. At the surface, atomic scale protrusions can form due to the lower degree of order. The TEM image is reproduced with permission of ref. 6 of the main text.

**Section S2. Equation adaptation for surface plasmon resonance quantification.**

The anti-Stokes/Stokes ratios are proportional to the width and frequency of the surface plasmon resonance as well as to the near-field temperature, which represent conditions to which the molecules are submitted in the near field. The method for extracting this information has originally been proposed by Brolo et al.^1^ in the context of single molecule SERS and is described in extended details elsewhere.^2-4^

Starting from the equations from Lombardi and Birke,^5^ the resonance model in plasmonic conditions has been developed by dos Santos et al.^3^ Interested readers are referred to the Supporting Information of this reference for an extensive justification of the model development. Here, we only present the development of equations that highlights the differences between the two approaches.

From Santos et al.,^3^ the ratio of anti-Stokes/Stokes measured in TERS, ρ*_TERS_*, can be normalized with the anti-Stokes/Stokes ratio of a reference bulk sample, ρ*_ref_*. In ref. 6, the normalization is made with an average SERS ratio, while here, bulk cysteine is used for this purpose.

$K=\frac{\rho_{TERS}}{\rho_{ref}}$ Eq. S1

with

$\rho_{TERS}= \frac{I_{AS, TERS}}{I_{S, TERS}}$ Eq. S2

$\rho_{ref}=\frac{I_{AS, ref}}{I_{S, ref}}$ Eq. S3

where S3 can be described by the Boltzmann distribution according to S4,

$\rho_{ref}= \frac{I_{AS, ref}}{I_{S, ref}}= \left( \frac{\bar{\upsilon}_{laser}- \bar{v}_{R}}{\bar{\upsilon}_{laser}+ \bar{v}_{R}} \right)^{3}exp\left( \frac{-hc\bar{\nu}_{R}}{kT} \right)$ Eq. S4

where ν*_laser_* and ν*_R_* are the frequency of the incident laser line and of the Raman mode, respectively, *h* is the Planck constant, *c* is the speed of light in vacuum, *k* is the Boltzmann constant and *T* is the temperature. *K_plasmon_* can be described by the product of two contributions, i.e. from the separate contributions of the electromagnetic enhancement (*K_EM_*) and of the chemical enhancement (*K_EC_*) (associated to the chemical effect), which can be described as:

$K_{EM}= \left[ \frac{\left( \bar{\nu}_{plasmon, EM}- \bar{\nu}_{laser}+ \bar{\nu}_{R} \right)^{2}+ \left( \frac{1}{2}Г_{plasmon, EM} \right)^{2}}{\left( \bar{\nu}_{plasmon, EM}- \bar{\nu}_{laser}- \bar{\nu}_{R} \right)^{2}+ \left( \frac{1}{2}Г_{plasmon, EM} \right)^{2}} \right]^{2}$ Eq. S5

and

$K_{EC}= \frac{\left( \bar{\nu}_{plasmon, CE}- \bar{\nu}_{laser}+ \bar{\nu}_{R} \right)^{2}+ \left( \frac{1}{2}Г_{plasmon,CE} \right)^{2}}{\left( \bar{\nu}_{plasmon,CE}- \bar{\nu}_{laser}- \bar{\nu}_{R} \right)^{2}+ \left( \frac{1}{2}Г_{plasmon,CE} \right)^{2}}$ Eq. S6

Such that

$K_{plasmon}= \left[ \frac{\left( \bar{\nu}_{plasmon, EM}- \bar{\nu}_{laser}+ \bar{\nu}_{R} \right)^{2}+ \left( \frac{1}{2}Г_{plasmon, EM} \right)^{2}}{\left( \bar{\nu}_{plasmon,EM}- \bar{\nu}_{plasmon}- \bar{\nu}_{R} \right)^{2}+ \left( \frac{1}{2}Г_{plasmon,EM} \right)^{2}} \right]^{2}\frac{\left( \bar{\nu}_{plasmon, CE}- \bar{\nu}_{laser}+ \bar{\nu}_{R} \right)^{2}+ \left( \frac{1}{2}Г_{plasmon,CE} \right)^{2}}{\left( \bar{\nu}_{plasmon,CE}- \bar{\nu}_{laser}- \bar{\nu}_{R} \right)^{2}+ \left( \frac{1}{2}Г_{plasmon,CE} \right)^{2}}$ Eq. S7

Where $\bar{\nu}_{plasmon}$,$\bar{\nu}_{laser}$, $\bar{\nu}_{R}$ are the plasmon resonance, laser energy, position of the vibrational mode (all in wavenumbers), respectively, and $Г$ is the damping parameter. The suffixes *EM* and *CE* refer to electromagnetic and chemical enhancement, respectively. In non-resonant conditions, after normalization with a bulk sample at room temperature and with the approximation that the polarizability tensor of a given mode is equivalent for the stokes and anti-stokes part, chemical enhancement vanishes (*K_EC_* = 1) and only the electromagnetic enhancement contributes to *K*, such that

$K_{plasmon}= \left[ \frac{\left( \bar{\nu}_{plasmon}- \bar{\nu}_{laser}+ \bar{\nu}_{R} \right)^{2}+ \left( \frac{1}{2}Г \right)^{2}}{\left( \bar{\nu}_{plasmon}- \bar{\nu}_{laser}- \bar{\nu}_{R} \right)^{2}+ \left( \frac{1}{2}Г \right)^{2}} \right]^{2}$ Eq. S8

From Eq. S4, two parameters are unknown, namely $\bar{\nu}_{plasmon}$ and $Г$. The plasmon resonance could still be determined by scanning the most likely possibilities of $\bar{\nu}_{res}$,$Г$couples.^3^

Here, since we have access to a series of vibrational modes for which the signal to noise ratio is satisfying (including in the anti-Stokes spectral region), Eq. S4 is expanded into a series.

$\sum_{i} K_{plasmon, i}= \sum_{i} \left( \frac{\left( \bar{\nu}_{plasmon}- \bar{\nu}_{laser}+ \bar{\nu}_{R,i} \right)^{2}+ \left( \frac{1}{2}Г \right)^{2}}{\left( \bar{\nu}_{plasmon}- \bar{\nu}_{laser}- \bar{\nu}_{R,i} \right)^{2}+ \left( \frac{1}{2}Г \right)^{2}} \right)^{2}$ Eq. S9

Where $K_{plasmon, i}$ is the experimentally determined anti-Stokes/Stokes ratio of each vibrational mode (*i*), $\bar{\nu}_{R,i}$.

By combining Eq. S2, S3, S4 and S9, the temperature parameter is included as well. It represents the temperature that the reference sample should have under TERS experimental conditions.

$\sum_{i} K_{plasmon, i}= \frac{\rho_{TERS, i}}{\left( \frac{\bar{\nu}_{laser}-\bar{\nu}_{R,i}}{\bar{\nu}_{laser}+\bar{\nu}_{R,i}} \right)^{3} exp\left( \frac{-{ch\bar{\nu}}_{R,i}}{kT} \right)}= \sum_{i} \left( \frac{\left( \bar{\nu}_{plasmon}- \bar{\nu}_{laser}+ \bar{\nu}_{R,i} \right)^{2}+ \left( \frac{1}{2}Г \right)^{2}}{\left( \bar{\nu}_{plasmon}- \bar{\nu}_{laser}- \bar{\nu}_{R,i} \right)^{2}+ \left( \frac{1}{2}Г \right)^{2}} \right)^{2}$ Eq. S10

Our results presented in Figure 2A indicate at least five band pairs acceptable for anti-Stokes/Stokes ratio determination, while Eq. S10 requires only three parameters, namely $\bar{\nu}_{plasmon}$, $Г$ and T to be solved. Using Mathematica software and the *NMinimized* function, the system converges to a single solution and makes use of the additional band pairs as well.

**Section S3. Stokes vs anti-Stokes symmetry**


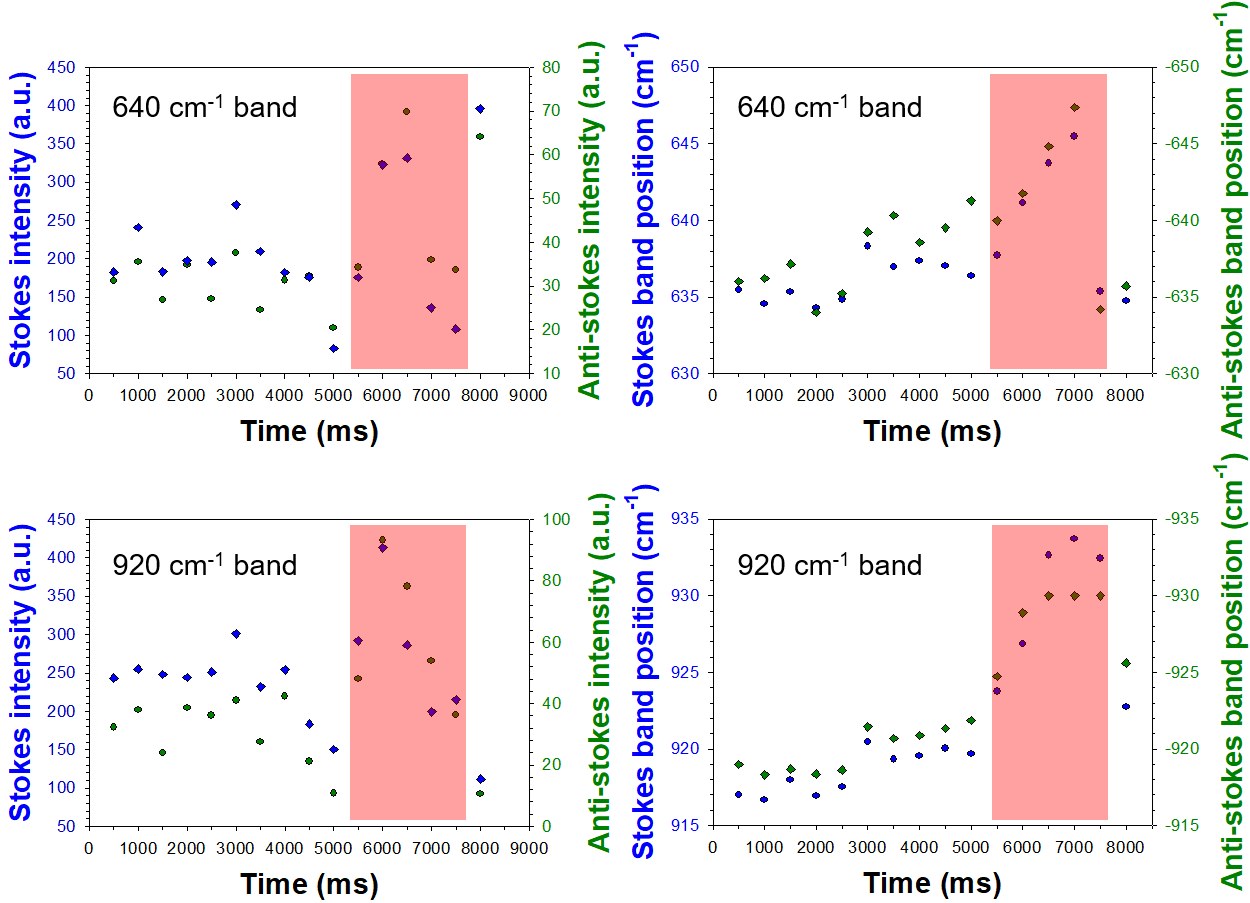


**Figure S5.** Example of Stokes (blue) vs anti-Stokes (green) intensity (left panels) and band position (right panels) evolution vs time for two *persistent lines* of the spectra presented in Figure 2. The highlighted region indicates the time frame in which *blinking lines* appeared in the spectra.

Other possibilities have been envisioned to justify sudden band appearance such as presence of contamination but have been ruled out based on a more detailed analysis of the band intensities and positions, as shown in Figure S5. An obvious alternative justification of our results would be that impurities could have diffused in and out of the hot spot region or be momentarily adsorbed on the tip at a location where the field enhancement is large, as reported by Ichimura et al. ^6^ in the context of time-dependent TERS measurements on adenine molecules. Here, this possibility is ruled out because the intensity of all *persistent lines* increases with the appearance of the *blinking lines* (left panels of Figure S5). This means that the “impurity” would participate at increasing the signal intensity of bands that were already present in addition to giving rise to new bands such that it would have to be of 1) of the same chemical nature and with the same spatial orientation with respect to the surface as the monolayer of 16-mepcaptodundecanoic acid, but 2) composed of a different chemical group leading to new and quite strong bands in the spectra, which is arguably unphysical.

The second possibility is that the tip induces local chemical changes in the hot spot regions and probes this change while (or just after) inducing it.^7^ This would lead to the simultaneous decrease of some bands and to the appearance of new ones, which is not observed here. Such charge transfer mechanism also modifies the plasmon position^3^ and an asymmetry of the *persistent lines* of the stokes vs anti-stokes spectral range would be observed. The symmetry of the Stokes and anti-Stokes bands intensity fluctuations (green vs blue points of the right panels of Figure S5) strongly indicates an absence of a significant plasmon position fluctuation.

Another justification for the shift of plasmon frequency with the appearance of blinking line could be that some “chemical effects” would affect the plasmon position due to the formation of a hybridized state between the plasmon and the molecule.^4^ Here, this would be attributed to additional changes in the electronic structure or of the metal/particle interactions due to the proximity of the plasmonic object.^8-9^ The resulting changes in the polarizability tensor should have an influence on the plasmon resonance quantified in Figure 2, but remains consistent with the model a sudden domination of nanoscale surface protrusions.

**Section S4. Gold plate characterization**

**Figure S6. A)** AFM topography image of a gold plate and **B)** height profile corresponding to the white line of A.

**References.**

1. Brolo, A. G.; Sanderson, A. C.; Smith, A. P., Ratio of the surface-enhanced anti-Stokes scattering to the surface-enhanced Stokes-Raman scattering for molecules adsorbed on a silver electrode. *Phys. Rev. B* **2004,** *69* (4), 045424.

2. dos Santos, D. P.; Andrade, G. F. S.; Brolo, A. G.; Temperini, M. L. A., Fluctuations of the Stokes and anti-Stokes surface-enhanced resonance Raman scattering intensities in an electrochemical environment. *Chem. Comm.* **2011,** *47* (25), 7158-7160.

3. dos Santos, D. P.; Temperini, M. L. A.; Brolo, A. G., Mapping the Energy Distribution of SERRS Hot Spots from Anti-Stokes to Stokes Intensity Ratios. *J. Am. Chem. Soc.* **2012,** *134* (32), 13492-13500.

4. dos Santos, D. P.; Temperini, M. L. A.; Brolo, A. G., Single-Molecule Surface-Enhanced (Resonance) Raman Scattering (SE(R)RS) as a Probe for Metal Colloid Aggregation State. *J. Phys. Chem. C* **2016,** *120* (37), 20877-20885.

5. Lombardi, J. R.; Birke, R. L., A Unified Approach to Surface-Enhanced Raman Spectroscopy. *J. Phys. Chem. C* **2008,** *112* (14), 5605-5617.

6. Ichimura, T.; Watanabe, H.; Morita, Y.; Verma, P.; Kawata, S.; Inouye, Y., Temporal Fluctuation of Tip-Enhanced Raman Spectra of Adenine Molecules. *J. Phys. Chem. C* **2007,** *111* (26), 9460-9464.

7. Kim, Y.; Kazuma, E., Mechanistic studies of plasmon chemistry on metal catalysts. *Angew. Chem. Int. Ed.* **2018,** *15* (58), 4801-4808.

8. Gieseking, R. L. M.; Lee, J.; Tallarida, N.; Apkarian, V. A.; Schatz, G. C., Bias-Dependent Chemical Enhancement and Nonclassical Stark Effect in Tip-Enhanced Raman Spectromicroscopy of CO-Terminated Ag Tips. *J. Phys. Chem. Lett.* **2018,** *9* (11), 3074-3080.

9. Morton, S. M.; Jensen, L., Understanding the Molecule−Surface Chemical Coupling in SERS. *J. Am. Chem. Soc.* **2009,** *131* (11), 4090-4098.
